# Inflammation, Immunosuppression, and Immunotherapy in Pancreatic Cancer—Where Are We Now?

**DOI:** 10.3390/cancers17091484

**Published:** 2025-04-28

**Authors:** Marta Fudalej, Kamila Krupa, Anna Badowska-Kozakiewicz, Andrzej Deptała

**Affiliations:** 1 Department of Oncological Propaedeutics, Medical University of Warsaw, 01-445 Warsaw, Poland; marta.fudalej@wum.edu.pl (M.F.); anna.badowska-kozakiewicz@wum.edu.pl (A.B.-K.); 2 Department of Oncology, National Medical Institute of the Ministry of the Interior and Administration, 02-507 Warsaw, Poland; 3 Students’ Scientific Organization of Cancer Cell Biology, Department of Oncological Propaedeutics, Medical University of Warsaw, 01-445 Warsaw, Poland; kamila.krupa@student.wum.edu.pl

**Keywords:** pancreatic cancer, immunotherapy, oncology, inflammation, immunosuppression, cancer vaccines

## Abstract

Pancreatic cancer (PC) is one of the most commonly diagnosed and deadliest neoplasms in the modern world. Over the past few years, the incidence of PC has risen with only a slight improvement in overall survival. Moreover, the improvement in survival is primarily driven by diagnoses in the localized stage of the disease, rather than by new treatment methods. The inflammatory process is a key mediator of PC development, yet PC is also one of the most immune-resistant tumors. Patients rarely benefit from monotherapy with immune checkpoint inhibitors; nevertheless, the latest biological findings on the complexity of the pancreatic tumor microenvironment might be translated into designing new clinical studies that combine various approaches to overcome single-agent immunotherapy resistance. On the other hand, focusing on inflammation may lead to the development of new inflammation-based prognostic markers for patients. This review aims to describe the current state of knowledge regarding the complex relationships between systemic and local inflammation, immune response, immunosuppression, and therapeutic options in PC.

## Simple Summary

The inflammatory process is one of the key mediators of pancreatic cancer (PC) development, yet at the same time, PC is one of the most immune-resistant tumors. Patients rarely benefit from monotherapy with immune checkpoint inhibitors; nevertheless, the latest biological findings on the complexity of the pancreatic tumor microenvironment might be translated into designing new clinical studies that combine various approaches to overcome single-agent immunotherapy resistance. On the other hand, focusing on inflammation may lead to the development of new inflammation-based prognostic markers for patients. This review aims to describe the current state of knowledge regarding the complex relationships between systemic and local inflammation, an immune response, immunosuppression, and therapeutic options in PC.

## 1. Introduction

Pancreatic cancer (PC) is one of the most commonly diagnosed and most deadly neoplasms in the modern world [1]. Over the past few years, the incidence of PC has risen with only a slight improvement in overall survival (OS), reaching 13% in 2024 [2]. A decade ago, the 5-year OS rate was only 6%; nevertheless, the improvement in survival is driven mainly by diagnoses at the localized stage of the disease, rather than by new treatment methods [3]. In the face of the complexity of patients’ conditions, multidisciplinary teams (comprising doctors of various specialties, dietitians, psychologists, and physiotherapists) should be established to care for them [4]. According to the newest National Comprehensive Cancer Network (NCCN) guidelines, treatment methods encompass surgery, systemic therapies, radiotherapy, and targeted therapy in some indications [5] The tumor is assessed as resectable, with no infiltration of major mesenteric blood vessels, i.e., the celiac axis, common hepatic artery, superior mesenteric artery, superior mesenteric vein, and portal vein. Systemic therapy is utilized in all stages of PC. The value of neoadjuvant treatment is still evolving, with no consensus about its influence on increased cure rates [6]. Preferred first-line regimens for adjuvant chemotherapy encompass modified FOLFIRINOX (mFOLFIRINOX) or gemcitabine with capecitabine as an alternative option [7,8]. In the locally advanced or metastatic stage of the disease, guidelines recommend mFOLFIRINOX or gemcitabine with albumin-bound paclitaxel [9,10]. Radiotherapy (RT) is not a first-line treatment for PC—it is only used in specific indications, such as sterilizing vessel margins or enhancing the likelihood of a margin-negative resection. Additionally, RT can be used to palliate pain and bleeding, or to alleviate local obstructive symptoms [11]. To enhance treatment possibilities, every patient should be tested for potentially actionable somatic findings such as fusions (ALK, NRG1, NTRK, ROS1, FGFR2, and RET), mutations (BRAF, BRCA1/2, KRAS, and PALB2), amplifications (HER2), microsatellite instability (MSI), mismatch repair deficiency (dMMR), or tumor mutational burden (TMB) [5]. BRCA-positive patients will benefit from platinum-based chemotherapy and maintenance with a Poly(ADP-ribose) Polymerase (PARP) inhibitor, such as Olaparib [12]. A programmed death receptor 1 (PD-1) inhibitor, Pembrolizumab, can be proposed as a second or later-line treatment for patients with MSI-high (MSI-H) or dMMR pancreatic tumors [13]. In patients with neurotrophic tropomyosin receptor kinase (NTRK) fusion, selective tyrosine kinase inhibitors such as larotrectinib or entrectinib are recommended; however, they remain inaccessible in many countries [10]. Nowadays, numerous studies concentrate on kinase inhibitors for molecularly defined subgroups of patients with PC. Protein kinase inhibitors are developed to suppress redundant signaling pathways and prevent resistance. Some ongoing studies encompass dual-targeting proteolysis-targeting chimeras (PROTACs) or nortopsentin analogs [14,15,16].

Immunotherapy has revolutionized the treatment of various malignancies and provided a deeper understanding of tumors’ biology. Several immunotherapies have been successfully applied to multiple cancers since the testing of PD-L1 and therapies targeting PD-1 or programmed cell death ligand 1 (PD-L1) inhibitors have become a standard of care in lung cancer and renal cell carcinoma [17]. On the one hand, PC is one of the most immune-resistant tumors; on the other, the inflammatory process is one of the key mediators of PC development [18,19]. Moreover, recent studies have demonstrated that systemic inflammation and immune response markers are correlated with the prognosis and treatment response of PC patients [20,21].

This review aims to describe the current state of knowledge regarding the complex relationships between systemic and local inflammation, the immune response, immunosuppression, and therapeutic options in PC.

## 2. Inflammation and Immunosuppression

In the 19th century, Virchow observed immune cells in neoplastic tissue and developed the concept of the role of chronic inflammation in cancer development. Since then, chronic inflammation has been linked to various stages of tumorigenesis, encompassing cellular transformation, promotion, survival, proliferation, invasion, angiogenesis, and metastasis. Many environmental causes of cancer and risk factors are associated with some form of chronic inflammation [22]. In a typical setting, inflammation is generally self-limiting, as the production of anti-inflammatory cytokines closely follows that of pro-inflammatory cytokines. The persistence of initiating factors or a failure of mechanisms required to resolve the inflammatory response leads to chronic inflammation [23]. Systemic diseases characterized by low-grade chronic inflammation, such as metabolic syndrome and diabetes mellitus, enhance the risk of cancer, particularly PC. Inflammatory cells simultaneously orchestrate inflammation, enhancing tumor development and governing the immune response against emerging tumor cells. Moreover, they might support the escape of tumor cells from immune control by favoring more immunosuppressive phenotypes and modifying their antigenic fingerprint. Tumor cell metabolism, primarily glucose metabolism, is both affected by and affects inflammatory cells [24]. Chronic inflammation can generate an immunosuppressive microenvironment that allows tumor formation and progression. The pro-inflammatory mediators facilitate crosstalk between different cells, creating a tumor-supporting, immunosuppressive microenvironment [25]. The pancreatic tumor microenvironment (TME) exhibits dynamic changes between inflammation and the immune response, primarily manifesting as inflammation-induced immunosuppression [26]. The immune response, controlling the identification and elimination of tumor cells, can be suppressed by chronic inflammation. Moreover, cytokines produced by inflammatory cells can inhibit the function of immune cells. It further simplifies the process by which cancer cells can proliferate and metastasize [27]. Although inflammation at the tumor site usually promotes cancer progression, it can be remodeled to acquire anti-tumor properties [28].

## 3. Inflammatory Markers

As previously mentioned, inflammation and the host’s ineffective immune response are primary hallmarks of PC development and progression. Many studies have reported that various pretreatment inflammatory markers may be prognostic factors in malignant tumors, including PC; nevertheless, which combination of these factors may be the most valuable remains undefined [20]. Moreover, they may be used to monitor the effectiveness of chemotherapy.

Some inflammatory markers include various settings of lymphocytes, neutrophils, platelets, and monocytes. One of them—the neutrophil-to-lymphocyte ratio (NLR)—was analyzed in several studies. The cut-off value of the NLR varied a lot, from two to five. Based on a meta-analysis of 43 cohort studies, including 8252 patients with PC, patients with a low NLR had a longer OS, disease-free survival (DFS), significantly smaller tumors, and lower grades, stages, and CA19-9 levels [29]. The underlying mechanism of the association between a high NLR and poor prognosis in patients with PC remains unclear. Neutrophils may promote the proliferation and metastasis of PC through multiple mechanisms, including mediating the epithelial–mesenchymal transition (EMT), inducing angiogenesis by secreting various cytokines, and creating an immunosuppressive microenvironment for PC [30,31]. In terms of lymphocytes, the relationship is far more complex, as there are controversies regarding the changes in T cells in PC patients and the contrasting responses of different T cell subtypes [29]. Nevertheless, a decreased lymphocyte count may reflect an insufficient immunological response to the tumor, as a reduction in lymphocytes might weaken the anti-tumor response and immune surveillance [24].

Another marker, the platelet-to-lymphocyte ratio (PLR), was assessed in the meta-analysis of 17 cohort studies, including 3182 patients with PC. The authors concluded that PLR could be a prognostic factor, as a low PLR was associated with a longer OS and progression-free survival (PFS) [32]. Similar to the NLR, the exact mechanism remains elusive. Platelets promote tumor growth, angiogenesis, metastasis, and cancer-associated thrombosis [33]. They also promote tumor cell adhesion to the microvascular endothelium and form a defense barrier around circulating tumor cells, helping them escape immune surveillance [34].

Hu et al. (2018) conducted a meta-analysis of the lymphocyte-to-monocyte ratio (LMR) as a prognostic indicator for patients with PC. The analysis included 11 cohorts comprising 2557 patients with PC. A low LMR was associated with an unfavorable OS, shorter DFS, and recurrence-free survival (RFS). A low LMR also correlated with higher CA19-9 and TNM stages [35]. In 2024, Li et al. examined whether LMR could serve as an indicator for predicting the prognosis of patients only with resectable PC. They confirmed the substantial predictive value of the LMR for OS; however, the LMR did not exhibit predictive significance for RFS [36]. Monocytes infiltrate tumors and differentiate into TAMs involved in tumor progression. As a result, an increased level of monocytes may express a higher tumor burden [37]. Furthermore, monocytes can inhibit antigen- and mitogen-induced lymphocyte proliferation, impair lymphocyte-dependent anti-tumor defenses, and suppress anti-tumor immunity [38]. Whether the circulating level of monocytes serves as a surrogate for the increased production of tissue macrophages remains an open question for further study.

The systemic immune–inflammation index (SII) is a combined biomarker based on the counts of lymphocytes, platelets, and neutrophils. It was first identified as a prognostic indicator for clinical outcomes of hepatocellular carcinoma (HCC) patients. Two literature review-based meta-analyses of the relationship between the SII and the prognosis of PC patients have shown that patients with higher SII scores have a worse prognosis [39,40]. Another parameter, the systemic inflammation response index (SIRI), is calculated using counts of neutrophils, lymphocytes, and monocytes. In the meta-analysis by Shen et al. (2024) [41] comprising seven studies and 1160 patients, a higher SIRI was associated with worse OS and PFS. Furthermore, prospective studies are needed to confirm whether the SII or SIRI is superior to the NLR or PLR alone in predicting survival in patients with PC.

Neumann et al. (2023) [42] developed a new score, termed the Inflammatory Benchmark Index (IBI). It combines the sum of neutrophils, monocytes, lymphocytes, and platelets, all divided by CRP. The IBI was highly significant in univariate and multivariate analyses of prognostic factors for OS in PC, independent of the tumor stage and PS. Interestingly, in this marker, unlike all the ones discussed before, lymphocytes are grouped together with monocytes, platelets, and neutrophils in the same numerator. This parameter takes CRP into account. In her PhD dissertation, Fudalej observed that among patients with PC and diabetes mellitus, a CRP level ≤ 5 mg/L was the strongest predictor of survival. Moreover, a lower level of another parameter, including CRP—the CRP-to-lymphocyte ratio (CLR)—was significantly associated with longer OS in the group of patients with PC and diabetes mellitus or hypertension [21,43].

The CRP/albumin ratio (CAR) is supposed to represent the potential inflammatory status of the body, as albumins can be involved in the inflammatory response. It may be associated with patients’ nutritional and septic status [44]. Hypoalbuminemia, as an exponent of malnutrition and cachexia, can exacerbate cancer-associated inflammation [45]. Most studies have focused on the CAR and analyzed its preoperative levels in patients with PC who are resectable. In one of them, the CAR was statistically superior to other prognostic markers, including the CLR, NLR, PLR, the modified Glasgow Prognostic Score (mGPS), and the Prognostic Index (PNI) [45]. In another study, a high CAR at postoperative day 14 was related to a high BMI, a large amount of intraoperative bleeding, and the presence of complications, all resulting in shorter OS and RFS [46]. Other authors have analyzed the CRP-to-prealbumin ratio, as some studies suggest that prealbumin may be a more sensitive marker than albumin for assessing the nutritional status [47]. In the study by Kwon et al. (2023), the CRP-to-prealbumin ratio was found to have a significant association with early recurrence after curative resection in patients with resectable PC [48]. Many studies have confirmed that the CAR may be a valuable predictor of recurrence or OS; however, as presented in Table 1, the cut-off values of the CAR vary considerably across different studies.

The CALLY index, derived from CRP, the serum albumin level, and total lymphocyte count, reflects the body’s immune, nutritional, and inflammatory status. It was initially established as a predictive marker in patients undergoing transarterial chemoembolization for HCC [49]. In the investigation of 3511 cancer-afflicted adults, the CALLY index demonstrated a linear and negative association with all-cause mortality, as well as mortality caused by cancer and cardiac conditions [50]. A recent study investigated the utility of the CALLY index in patients undergoing curative surgical treatment for PC. It proved that a low CALLY score reflected a higher risk of complications and mortality due to a weaker immune function, poorer nutritional status, and higher systemic inflammation [51]. In the study by Kawahara et al. (2024), a low CALLY index was significantly associated with a larger tumor size, metastatic lymph nodes, a worse pathological TNM stage, older age, and a low administration rate of adjuvant chemotherapy [52]. Another study examining the preoperative value of the CALLY index in PC patients reported its superior prognostic ability for DFS and OS compared to other scores, including the Geriatric Nutritional Risk Index (GNRI), SII, Controlling Nutritional Status (CONUT score), NLR, and PLR [53]. The CALLY index can be used as an objective indicator reflecting the tumor’s biological malignancy and the patient’s condition. The authors suggest that the CALLY index may be valuable for guiding surgical decision making. Patients with a low CALY index may benefit from initial treatment with neoadjuvant chemotherapy, accompanied by intensive nutritional status enhancement and a reduction in inflammation [53].

As discussed above, various markers or indexes may be implemented to predict the outcomes of patients with PC; nevertheless, there are still more questions than answers. Firstly, most of the presented studies are retrospective, encompassing different stages of PC and other treatment methods. Non-specific processes, such as trauma, infections, or immune-related diseases, may affect cell counts. The same parameter had no uniform threshold; the cut-off values varied significantly between studies. We believe that conducting a well-planned prospective study with a large sample, comparing different indexes, will provide a clear answer about the best new inflammation-based prognostic marker for patients with PC.

**Table 1:** Analysis of studies examining various parameters for predicting PC prognosis.

Parameter	How Counted	Cut-Off Value	No of Patients	Time of Measurement	Association Between High Parameters and Outcomes	Ref.
CAR	CRP (mg/L) ÷ albumin (g/dL)	0.18	595	Pretreatment Post-chemotherapy	Shorter OS	[54]
		0.06	163	Preoperative	Shorter OS and DFS	[45]
		0.34	142	14 postoperative days	Shorter OS and RFS	[46]
		3.85	302	Pretreatment	Shorter OS and PFS	[55]
		0.09	143	Preoperative	Shorter OS	[56]
		0.03	113	Preoperative	Shorter OS and RFS	[44]
		0.4	1294	Pretreatment	Shorter OS	[42]
CRP/ pre-albumin	CRP (mg/dl) ÷ prealbumin (mg/dL)	1.3	20	Preoperative	Shorter RFS	[48]
CALLY	[albumin (g/L) × lymphocyte count] ÷ [CRP (mg/L) × 10^4^]	1.03	121	Preoperative	Lower rates of postoperative complications, longer OS	[51]
		3.00	307		Longer OS and DFS	[53]
		1.90	461		Longer OS and RFS	[52]
SII	[platelet count × neutrophil count] ÷ lymphocyte count	400–900	2132 (meta-analysis), 1749 (meta-analysis)	Pretreatment	Shorter OS, DFS, and PFS	[39,40]
SIRI	[neutrophil count × monocyte count] ÷ lymphocyte count	0.69–2.35	1160 (meta-analysis)	Pretreatment	Shorter OS and PFS	[41]
LMR	lymphocyte count ÷ monocyte count	2.05–4.62	2557 (meta-analysis)	Pretreatment	Longer OS, DFS, RFS	[35]
		1.60–5.00	4019 (meta-analysis)	Preoperative	Longer OS	[36]
NLR	neutrophil count ÷ lymphocyte count	2.00–5.00	8252 (meta-analysis)	Pretreatment	Shorter OS and DFS	[29]
PLR	platelet count ÷ lymphocyte count	126–300	3182 (meta-analysis)	Pretreatment	Shorter OS and PFS	[32]
IBI	[lymphocyte count + monocyte count + neutrophil count + platelet count] ÷ CRP (mg/L)	30	1294	Pretreatment	Longer OS	[42]

Abbreviations: CAR—CRP-to-albumin ratio, OS—overall survival, DFS—disease-free survival, PFS—progression-free survival, RFS—recurrence-free survival, CALLY—CRP-albumin-lymphocyte index, SII—immune–inflammation index, SIRI—systemic inflammation response index, LMR—lymphocyte-to-monocyte ratio, NLR—neutrophil-to-lymphocyte ratio, PLR—platelet-to-lymphocyte ratio, IBI—inflammatory benchmark index.

## 4. Immunotherapy

Physiologically, when tumor cells invade healthy tissue, the immune system should recognize and eliminate them. Unfortunately, cancerous cells can evade the immune system through different immune escape mechanisms. The main mechanisms encompass (1) recruiting suppressor immune cells (myeloid-derived suppressor cells (MDSCs), regulatory T-cells (Tregs), and cytokines) to form a suppressive immune microenvironment; (2) down-regulating surface antigen expression resulting in immunogenicity decrease; (3) suppressing T-cell activity by up-regulating the immune checkpoint on the surface; and (4) releasing acidic and toxic metabolites which inhibit the activity of immune cells in the TME [57]. Immunotherapy aims to boost the natural defenses of the immunological system to eliminate malignant cells. This kind of treatment is a breakthrough and has revolutionized the field of oncology [58]. The immune responses explicitly targeting cancer cells, triggered by immunotherapy, differ from those stimulated by tumor-directed therapies. Moreover, the response to immunotherapy can endure for a prolonged period even after treatment is discontinued [59].

### 4.1. Current Immunotherapy Options in PC

MSI-H or dMMR is detected in approximately 1% of patients with PC. It is typically associated with Lynch syndrome, but may also occur sporadically due to promoter methylation or somatic mutations [60]. In 2017, based on five uncontrolled, multicohort, multicenter, and single-arm clinical trials, the U.S. Food and Drug Administration (FDA) granted accelerated approval to pembrolizumab for adult and pediatric patients with unresectable or metastatic MSI-H or dMMR solid tumors that have progressed after previous treatment and for whom there are no satisfactory alternative options. In 2023, the approval was converted to full, supported by findings from the multicenter, non-randomized, open-label, multicohort, phase 2 KEYNOTE-158, KEYNOTE-164, and KEYNOTE-051 trials. Pembrolizumab is an anti-PD-1 receptor antibody that blocks its interaction with PD-L1 and PD-L2 [61,62]. According to the latest NCCN guidelines, pembrolizumab is recommended for patients with advanced MSI-H/dMMR PC for both first-line and subsequent treatment, provided no prior immunotherapy has been implemented [5]. ESMO recommends pembrolizumab as a second or later-line treatment in this cohort [10]. NCCN also includes Dostarlimab-gxly as a subsequent treatment option (with no prior immunotherapy) for patients with MSI-H or dMMR locally advanced, metastatic, or recurrent PC and any performance status (PS) [63] and nivolumab/ipilimumab as a subsequent therapy option for patients with a high tumor mutational burden (TMB-H) and with good or intermediate PS [64], however, with category 2B of recommendation strength.

### 4.2. Why Is Single-Agent Immunotherapy Not Effective in PC?

**Figure 1:**
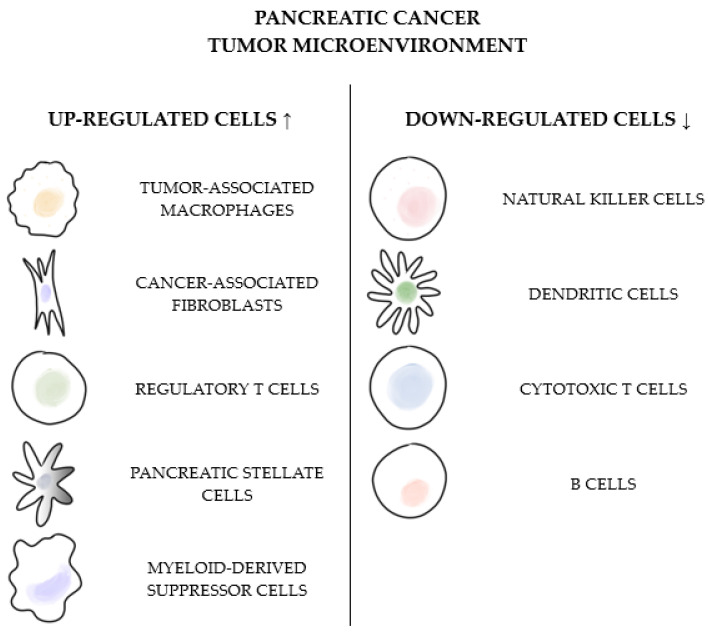
The graphical summary of up- and down-regulated cells in the pancreatic cancer tumor microenvironment.

PC patients rarely benefit from treatment with immune checkpoint inhibitors (ICI) due to the properties of the highly immunosuppressive TME; pancreatic tumors are often considered “cold tumors”. The low expression of the major histocompatibility complex (MHC) class I molecules on PC cells inhibits T-cell activation, as they are activated by an interaction with antigens presented by MHC I molecules on antigen-presenting cells [65]. Up-regulated proteins, facilitating immunosuppresion and tumor progression, encompass Tregs, tumor-associated macrophages (TAMs), MDSCs, cancer-associated fibroblasts (CAFs), and pancreatic stellate cells (PSCs) (Figure 1) [66].

PSCs are one of the key components of the TME. They release various cytokines and chemokines, such as IL-6, C-X-C motif chemokine ligand (CXCL)12, or CXCL10. CXCL12 sequesters CD8 T-cells and hinders their antitumor activity, while CXCL10 contributes to the recruitment of Tregs. Tregs induce immune suppression through various mechanisms, such as secreting immunosuppressive cytokines or inhibiting dendritic cell maturation. What is more, permanently expressed on Tregs, cytotoxic T-lymphocyte-associated protein 4 (CTLA-4) plays a significant role in suppressing antigen-presenting cells [65]. On the other hand, IL-6 promotes the migration of MDSCs to the TME. MDSCs further suppress T-cell proliferation by depleting L-arginine and L-tryptophan. Also, the production of reactive oxygen species (ROS) and reactive nitrogen species (RNS) by MDSCs leads to a decrease in the reactivity of MHC I molecules with cytotoxic T-cells [67]. PC presents a dense layer of cancer-associated fibroblasts (CAFs) surrounding malignant cells. CAFs actively crosstalk with cancer cells within the TME and contribute to drug resistance. One of the hallmarks of the physical and immunological barriers of PC is a prominent desmoplastic reaction. The dense and fibrous connective tissue comprising cellular and non-cellular components prevents immune cells from infiltrating the tumor [68]. Down-regulated cells include previously mentioned CD8+ cytotoxic T lymphocytes, natural killer (NK) cells, dendritic cells (DCs), and some phenotypes of B-cells [69]. The poor infiltration of effector T cells leads to the insufficient action of immune checkpoint inhibitors. The hypoxic state of the TME also leads to resistance to immunotherapy, as hypoxia-induced HIF-1α can promote PD-L1 expression in cancer cells and suppress the immune effect. The TME under hypoxia provides survival conditions for tumor cells and obstructs the effect of anti-tumor drugs by hindering drug delivery [70]. Last but not least, PC is characterized by a low mutation burden in most cases; thus, fewer tumor-specific neoantigens are available to trigger an immune response, making checkpoint inhibitors even less effective [71].

### 4.3. Clinical Trials

**Figure 2:**
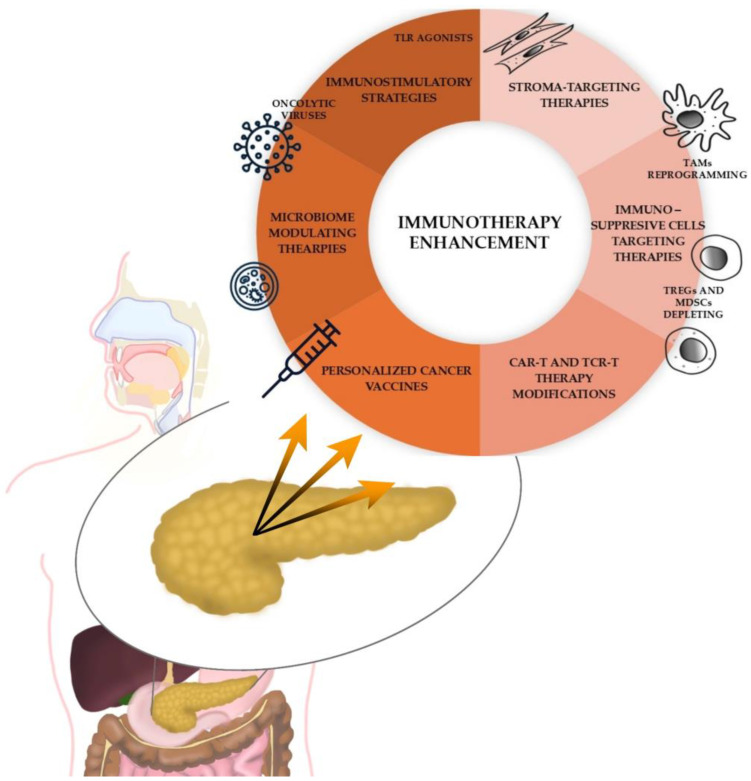
Different strategies for enhancing immunotherapy potential in pancreatic cancer.

As shown in Figure 2, various approaches are employed to overcome resistance to immunotherapy. One of them is combining immunostimulatory strategies. The DURIPANC trial combines an ICI Durvalumab with a Toll-like receptor (TLR-3) agonist (NCT05927142). TLR-3 agonists are hypothesized to induce the production of pro-inflammatory cytokines, including type I interferon (IFN-I), increase dendritic cell maturation, and the cross-priming of naive cytotoxic CD8+ T cells while eliminating the attraction of Tregs [72]. Preliminary data from this study demonstrated early disease control and safety in patients with late-stage PC [73]. Another study combines Pembrolizumab with dipeptidyl peptidase (DPP) and fibroblast activation protein (FAP) inhibitor—Talabostat (NCT05558982). Talabostat is intended to upregulate immune responses against cancer by increasing cytokine and chemokine production, thereby enhancing tumor-specific T-cell immunity. Moreover, high-affinity interactions involving FAP stimulate the anti-tumor activity of neutrophils, macrophages, and natural killer cells [74]. In the preliminary findings, BXCL701 (talabostat) plus pembrolizumab was well-tolerated and showed early signs of potential clinical activity in patients with metastatic PC refractory to chemotherapy [75]. Other approaches combine multi-kinase inhibitors, such as anlotinib, cabozantinib, or levantinib, with anti-PD-1 immunotherapy and chemotherapy (NCT06051851, NCT05052723, and NCT04887805). Multi-kinase inhibitors work synergistically with ICI by depleting the TME. They reduce immunosuppressive MDSCs and Tregs, enhance anti-tumor macrophage activity, and inhibit tumor growth and dissemination [76,77]. Some studies design immune-based therapies against KRAS or BRCA mutations (NCT06015724 and NCT05093231). By reversing some KRAS-initiated immunosuppressive changes, KRAS inhibitors make tumors more sensitive to immunotherapy [78]. Several ongoing clinical trials that combine immunotherapy with other therapeutic strategies are presented in Table 2.

### 4.4. Vaccine-Based Immunotherapy

Therapeutic cancer vaccines aim to generate long-lived, tumor-specific, functional T-cells that destroy cancer cells [79]. Vaccination is mainly considered in PC in the adjuvant setting due to the minimized tumor disease burden and TME immunosuppression, which may, in effect, improve clinical efficacy [18]. Types of cancer vaccines in PC include whole-cell, dendritic cell, peptide-based, mRNA-based, and viral-vector vaccines [80].

One of the most intensively studied whole cancer cell vaccines is GVAX (an allogenic, human granulocyte macrophage-colony-stimulating factor (GM-CSF)-secreting whole-cell PC vaccine) [59]. GVAX, when administered with cyclophosphamide (Cy) to deplete Tregs, has been shown to induce T-cell infiltration in TME. Moreover, vaccine therapy induced PD-L1 expression in both tumor epithelial cells and myeloid cells within the TME. These observations resulted in the design of studies that combined GVAX with ICI [81,82]. One of them—a platform trial of neoadjuvant and adjuvant antitumor vaccination with a PD-1 antagonist (nivolumab) and CD137 agonist (urelumab)—proved that this triplet arm is safe, increased intratumoral activated, cytotoxic T cells, and demonstrated a potentially promising efficacy signal in resectable pancreatic ductal adenocarcinoma (PDAC). Nevertheless, there was no DFS benefit to adding a PD-1a antagonist compared to GVAX alone. The triple regimen was associated with a marginally significant improvement in DFS compared to GVAX alone. The authors emphasized that this study was underpowered to achieve statistical significance, and further studies involving larger patient groups are required [83]. Another ongoing Phase 1 and 2 trial focuses on the CD137 agonist AGEN2373, combined with balstilimab (anti-PD-1), in combination with GVAX (Arm 1) or mKRASvx (Arm 2) (NCT06782932).

DC vaccines promote the activation of cytotoxic CD8+ T cells by presenting antigens to CD4+ and CD8+ T cells and secreting cytokines such as IL-15, IL-12, IFN-γ, and TNF [84]. Although studies on monotherapy with DC vaccines have shown no improvement in survival, DC vaccines have induced cytotoxic T cells and enhanced cytotoxic cytokine signaling, thereby suggesting that they may strengthen immunity, but require alternative combination therapeutics to improve outcomes [85]. This ongoing study examines the DC vaccine loaded with personalized peptides in combination with adjuvant chemotherapy (mFOLFIRINOX or gemcitabine/capecitabine), followed by nivolumab (NCT04627246). Another active study focuses on delivering autologous DC loaded with pancreatic adenocarcinoma lysate plus mRNA as an adjuvant therapy following the completion of standard chemotherapy (NCT04157127).

Peptide-based vaccines can induce immunity against specific antigenic epitopes derived from the vaccinated proteins or peptides expressed in cancer cells [80]. Neoantigens or tumor-specific antigens are applied to stimulate an immune response. They might be identified via the genetic testing of samples from patients’ biopsies to deliver the most suitable personalized vaccine [86]. Peptide-based vaccines present tumor-specific antigen peptides to MHC molecules to elicit the production of specific and long-term memory T-cells against cancer. MHC class I cells stimulate CD8+ cytotoxic T cell responses, while a small proportion of MHC class II cells stimulate CD4+ helper cells. CD4+ T-cells support the anticancer effect of CD8+ T-cells through enhancing the antigen presentation and releasing cytokines such as INF-γ and IL-2. [87]. One of the key peptides targeted in PC is mutant KRAS, as activating KRAS mutations are detected in over 90% of PDAC cases and implicated in tumor initiation and progression [88]. Recently, the AMPLIFY-201 Phase 1 study on patients with PC who are positive for minimal residual mKRAS disease (ctDNA and/or a serum tumor antigen) after locoregional treatment showed promising results. It examined the ELI-002 2P cancer vaccine, which enhances lymph node delivery and the immune response. The vaccine consists of the amphiphile (Amph) modification of G12D and G12R mutant KRAS (mKRAS) peptides (Amph-Peptides-2P) together with the CpG oligonucleotide adjuvant (Amph-CpG-7909). The therapy was well-tolerated, induced significant T-cell responses, and resulted in biomarker clearance and improved RFS [89]. These positive findings on the 2-peptide formulation of ELI-002 led to the design of another ongoing Phase 1 and 2 study on ELI-002 7P, which includes seven Amphipathic-modified KRAS and NRAS peptides: G12D, G12R, G12V, G12A, G12C, G12S, and G13D (Amph-Peptides 7P) (NCT05726864). Another promising, still recruiting study investigates the combination of the KRAS peptide vaccine with Ipilimumab and Nivolumab for patients with resected PDAC after neoadjuvant and/or adjuvant chemotherapy and/or radiation. The designed vaccine consists of polyinosinic–polycytidylic acid synthetic long peptides corresponding to six common mKRAS subtypes: G12D, G12R, G12V, G12A, G12C, and G13D (NCT04117087) [90].

mRNA vaccines function by delivering a single-stranded molecule encoding viral proteins or neoantigens. The molecule is incorporated into the cytoplasm of antigen-presenting cells and further translated into neoantigen proteins. Subsequently, mature antigen-presenting cells are transported to lymph nodes, generating a remarkable humoral and cellular immune response [91]. One of the most widely studied mRNA cancer vaccines is autogene cevumeran, an individualized mRNA neoantigen vaccine containing up to 20 primary histocompatibility complex class I (MHC I) and MHC class II (MHC II) restricted neoantigens in lipoplex nanoparticles, delivered intravenously. The study by Rojas et al. (2023) reported preliminary evidence that adjuvant autogene cevumeran, in combination with atezolizumab and mFOLFIRINOX, induced substantial T-cell activity in patients with surgically resected PDAC that correlated with delayed recurrence [92]. Based on these findings, a phase II, open-label, multicenter, randomized trial was designed to compare adjuvant autogene cevumeran with atezolizumab and mFOLFIRINOX versus mFOLFIRINOX alone (NCT05968326).

Viral-vector vaccines contain modified viruses that deliver cancer-specific genes or antigens, stimulating the immune system [93]. One is CRS-207, a live, attenuated, double-deleted Listeria monocytogenes (LADD) engineered to stimulate an immune response to mesothelin. The results from the ECLIPSE study showed that the combination of Cy/GVAX and CRS-207 did not improve survival compared to chemotherapy [94]. In further trials, adding nivolumab to Cy/GVAX plus CRS-207 did not improve the OS; however, objective responses and immunologic changes in the TME were evident [95]. The ongoing Phase 2 clinical trial is evaluating the combination of CRS-207 with pembrolizumab, ipilimumab, and tadalafil in patients with metastatic PDAC (NCT05014776). The pre-clinical study by Gross et al. (2024) demonstrated that phosphodiesterase type 5 (PDE5) inhibition with tadalafil, combined with vaccine-based immunotherapy, promoted pro-inflammatory states in myeloid cells, activated T cells, and enhanced crosstalk between myeloid cells and T cells [96].

Most vaccine therapy clinical trials have not demonstrated dramatic improvements in survival. However, most of these studies demonstrate signals of increased cancer antigen-specific T-cell responses after vaccination and potent ICI enhancement [85].

## 5. Conclusions

The inflammatory process is a key mediator of PC development, yet PC is also one of the most immune-resistant tumors. A plethora of various aspects relating to the involvement of inflammatory and anti-inflammatory processes are being investigated; nevertheless, none of them have turned out to be a panacea for PC. Patients with PC rarely benefit from monotherapy with immune checkpoint inhibitors, as indications for the use of anti-PD-1 antibodies are limited to only 1% of PC cases that exhibit dMMR or MSI-H. Nevertheless, the latest biological findings on the complexity of the TME may be translated into designing new clinical studies that combine various approaches to overcome resistance to single-agent immunotherapy. Some of them include combining immunostimulatory strategies or using specifically developed cancer vaccines. Another aspect is that focusing on inflammation may lead to new inflammation-based prognostic markers for patients. Establishing new indices or markers from the blood samples tested before the first course of chemotherapy may result in more accurate treatment choices.

## Figures and Tables

**Table 2:** Ongoing clinical trials combining immunotherapy with other agents.

Combination	Phase	Indication	Identifier
DARATUMUMAB (anti-CD38) + KRAS vaccine + NIVOLUMAB (anti-PD-1)	Phase II	Advanced PDAC—second-line treatment * Mutant KRAS in codon 12 (12A, C, D, R, S, V) or 13D	NCT06015724
DURVALUMAB (anti-PD-1) + RINTATOLIMOD (TLR-3 agonist)	Phase I, Phase II	Metastatic PDAC—first-line treatment	NCT05927142
DURVALUMAB (anti-PD-1) + OLECLUMAB (anti-CD73)	Phase II	Resectable PDAC—treatment before the surgery	NCT06060405
PEMBROLIZUMAB (anti-PD-1) + TALABOSTAT (DPP8/9 and FAP inhibitor)	Phase II	Metastatic PDAC—second-line treatment	NCT05558982
PENPULIMAB (anti-PD-1) + ANLOTINIB (multi-targeting TKI) + chemotherapy (gemcitabine + nab-paclitaxel)	Phase II	Metastatic PC—first-line treatment	NCT06051851
BOTENSILIMAB (CTLA-4 inhibitor) + chemotherapy (gemcitabine + nab-paclitaxel)	Phase II	Metastatic PDAC—second-line treatment	NCT05630183
TADALAFIL (PDE5 inhibitor) + PEMBROLIZUMAB (anti-PD-1) + IPILIMUMAB (CTLA-4 inhibitor) + CRS-207 (Listeria monocytogenes vaccine)	Phase II	Metastatic PDAC—second- or later-line treatment	NCT05014776
AVELUMAB (anti-PD-L1) + PEPINEMAB (SEMA4D inhibitor)	Phase I, Phase II	Metastatic PDAC—second-line treatment	NCT05102721
Chemotherapy + SBRT + NIVOLUMAB (anti-PD-1) + IPILIMUMAB (CTLA-4 inhibitor)	Phase I	Metastatic PDAC—first-line treatment	NCT05088889
Neoadjuvant chemotherapy (mFOLFIRINOX + PEMBROLIZUMAB (anti-PD-1) + surgery + adjuvant chemotherapy (mFOLFIRINOX) + PEMBROLIZUMAB	Phase II	Resectable PDAC—treatment before and after the surgery	NCT05132504
CABOZANTINIB (multi-targeting TKI) + PEMBROLIZUMAB (anti-PD-1)	Phase II	Metastatic PDAC—second- or later-line treatment	NCT05052723
LEVANTINIB (multi-targeting TKI) + PEMBROLIZUMAB (anti-PD-1)	Phase II	Metastatic/Unresectable PDAC—maintenance (PR/SD after 16 weeks of 1st- or 2nd-line treatment)	NCT04887805
NIRAPARIB (PARP inhibitor) + DOSTARLIMAB (anti-PD-1)	Phase II	Metastatic PDAC—second- or later-line treatment * germline or somatic mutations BRCA1/2, PALB2, BARD1, RAD51C, or RAD51D	NCT04493060
OLAPARIB (PARP inhibitor) + PEMBROLIZUMAB (anti-PD-1)	Phase II	Metastatic PDAC * dMMR or TMB > 4 Mutations/Mb	NCT05093231
XH001 (neoantigen cancer vaccine) + IPILIMUMAB (CTLA-4 inhibitor) + chemotherapy (gemcitabine + capecitabine)	Phase I, Phase II	Resectable PDAC—treatment after the surgery	NCT06353646
Irreversible electroporation + PEMBROLIZUMAB (anti-PD-1)	Phase I	Locally advanced unresectable PC—treatment after chemotherapy and ablative stereotactic magnetic resonance image-guided adaptive radiation therapy	NCT06378047
ANLOTINIB (multi-targeting TKI) + BENMELSTOBART (anti-PD-L1) + chemotherapy (gemcitabine + nab-paclitaxel)	Phase II	Metastatic PC—first-line treatment	NCT06621095
SBRT + BOTENSILIMAB (Fc-enhanced anti-CTLA-4) + BALSTILIMAB (anti-PD-1)	Phase II	Metastatic PDAC—second- or later-line treatment	NCT06843551
AGEN1423 (anti-CD73-TGF-β-trap) + BOTENSILIMAB (Fc-enhanced anti-CTLA4)	Phase II	Advanced PDAC—second- or later-line treatment	NCT05632328

Abbreviations: PDAC—pancreatic ductal adenocarcinoma, PD-1—programmed cell death protein 1, TLR-3—toll-like receptor 3, DPP—dipeptidyl peptidases, FAP—fibroblast activation protein, TKI—tyrosine kinase inhibitor, PDE5—phosphodiesterase 5, CTLA-4—cytotoxic T-lymphocyte–associated antigen 4, SEMA4D—semaphorin 4D, PR—partial response, SD—stable disease, PARP—poly-ADP ribose polymerase, dMMR—mismatch repair deficiency, TMB—tumor mutational burden, SBRT—stereotactic body radiation therapy. *—required mutations.

## Abbreviations

The following abbreviations are used in this manuscript:

PC	Pancreatic cancer
OS	Overall survival
NCCN	National Comprehensive Cancer Network
mFOLFIRINOX	Modified FOLFIRINOX
RT	Radiotherapy
MSI	Microsatellite instability
MSI-H	High microsatellite instability
dMMR	Mismatch repair deficiency
TMB	Tumor mutational burden
PARP	Poly(ADP-ribose) Polymerase
NTRK	Neurotrophic tropomyosin receptor kinase
PD-L1	Programmed cell death ligand 1
TME	Tumor microenvironment
MDSCs	Myeloid-derived suppressor cells
Tregs	Regulatory T-cells
FDA	U.S. Food and Drug Administration (FDA)
ESMO	European Society for Medical Oncology
PS	Performance status
TMB-H	High tumor mutational burden
ICI	Immune checkpoint inhibitor
TAMs	Tumor-associated macrophages
TLR	Toll-like receptor
IFN-I	Type I Interferon
DPP	Dipeptidyl peptidase
FAP	Fibroblast activation protein
NLR	Neutrophil-to-lymphocyte ratio
DFS	Disease-free survival
EMT	Epithelial-to-mesenchymal transition
PLR	Platelet-to-lymphocyte ratio
PFS	Progression-free survival
LMR	Lymphocyte-to-monocyte ratio
RFS	Recurrence-free survival
SII	Immune–inflammation index
SIRI	Systemic inflammation response index
IBI	Inflammatory benchmark index
CAR	CRP/albumin ratio
mGPS	Modified Glasgow prognostic score
PNI	Prognostic index
CALLY index	CRP-albumin-lymphocyte index
GNRI	Geriatric nutritional risk index
CONUT status	Controlling nutritional status
PDAC	Pancreatic ductal adenocarcinoma
PDE5	Phosphodiesterase type 5
CXCL	C-X-C motif chemokine ligand
MHC	Major histocompatibility complex
CAF	Cancer-associated fibroblasts
PSC	Pancreatic stellate cell
ROS	Reactive oxygen species
RNS	Reactive nitrogen species
NK	Natural killer
DC	Dendritic cells
